# Breast reconstruction after nipple/areola-sparing mastectomy using cell-enhanced fat grafting

**DOI:** 10.3332/ecancer.2009.116

**Published:** 2009-06-05

**Authors:** C Calabrese, L Orzalesi, D Casella, L Cataliotti

**Affiliations:** Breast Surgery Unit, Department of Oncology, University Hospital of Careggio, Florence, Italy

## Abstract

**Background:**

The success of fat grafting in breast reconstruction depends on fat retention. The use of stem-cells-enriched fat graft is an alternative method for graft stability.

**Case report:**

A case of nipple-areola sparing mastectomy double stage reconstruction with the use of stem cells enhanced fat graft is reported.

**Conclusions:**

Fat grafting is growing as a new and promising tool in reconstruction following nipple and areola sparing mastectomies as a way to restore a sufficient and reliable subcutaneous space in the mastectomy flap. This combined with an anatomical gel implant offers an integrated system of achieving a natural shaped breast.

## Introduction

Through preservation of the areola–nipple (A/N) complex, the nipple–areola sparing mastectomy (NASM) serves to reduce the negative psychological impact on women without increasing the incidence of local relapse [1,2]. For many women, the A/N complex is a distinctive element of the breast and plays a key role defining their body image [3]. NASM is particularly indicated in mid- to small-breasted women with a neoplasia, which prohibits a conservative surgical approach due to a disadvantageous relationship between breast dimensions and tumour size [1].

Actually, NASM is contraindicated in cases of the following:

1. involvement of the skin above the neoplastic area;
2. evidence of neoplastic infiltration of the A/N complex (nipple retraction or haematic secretion);
3. distance between the neoplasia and the A/N complex of less than 10 mm;
4. tumour multiplicity;
5. relapse in case of previous breast tumour;
6. suspicious axillary adenopathy;
7. primary chemotherapeutic treatment.

With NASM, the mammary gland is removed as well as the skin above the neoplasia when necessary. The incision can be made either inside the A/N complex (reverse omega), in the superior-outer quadrant with a radial course, or in the infra-mammary fold. An immediate reconstruction is usually performed with a tissue expander, which is replaced after six months by a definitive prosthesis with eventual symmetrization of the contralateral breast.

## Clinical case

**Figure 1:**
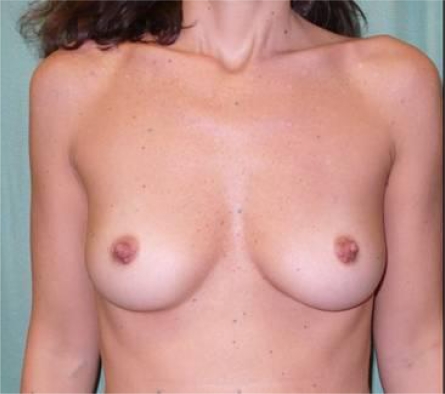
A 37-year-old patient before bilateral NASM (preoperative view)

The patient is a 37-year-old woman with a positive genetic test for BRCA1 and a nodular neoformation in the superior-outer/superior-inner quadrant of the right breast (x-ray mammograph: R5, breast ultrasound U5, AGB B5) Figure 1.

She underwent bilateral NASMs with a right sentinel lymph node biopsy on 20 June 2007.

Final histological examination documented infiltrating ductal carcinoma on the right side (NOS-G3 peritumoural haematic/lymphatic vascular invasion absent) with a minimum distance between the tumour and the nipple margin greater than 10 mm.

The sentinel lymph node biopsy was negative pT1c, psN0(i-).

The biological characterization of the neoplasm was the following: ER neg-PGr neg-ki67 40%, c-erb-B2 neg, score 0. Histological examination of the tissue from the left breast demonstrated micro-fibroadenoma intracanalicolare (QSE) in the background of fibrocystic mastopathy.

**Figure 2:**
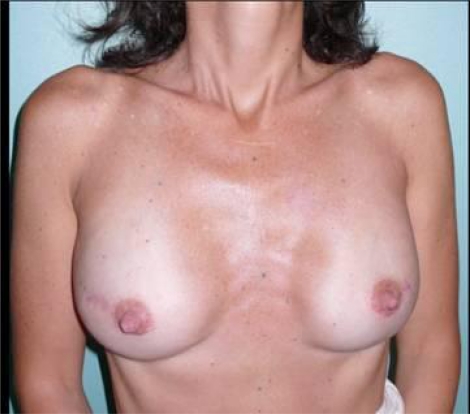
Post-operative view after bilateral NASM and immediate reconstruction with a bilateral 450 cc tissue expander

Immediately following the mastectomies, a bilateral 450 cc tissue expanders were positioned in a submuscular pocket Figure 2

The post-operative course was free of complication. In August 2007, the patient underwent prophylactic bilateral oophorectomies. On 28 January 2008, the patient underwent the second stage of the breast reconstruction. A partial bilateral capsulotomy was followed by a careful injection of cell-enhanced autologous fat into the subcutaneous areas. A total of 85 cc of cell-enhanced fat graft was injected on the left breast and 90 cc on the right. The tissue was obtained through a syringe-based liposuction of 355 cc of adipose tissue from abdomen and external thighs, which was performed at the beginning of the case. The tissue was then processed using an automated device (Celution 800/CRS-Cytori) to produce a cell-enhanced fat graft.

**Figure 3:**
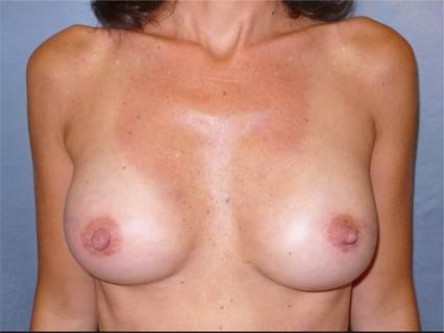
Post-operative view after second stage of breast reconstruction with 370-cc bilateral silicone gel implants and injection of cell-enhanced autologous fat (85 cc in the left side and 90 in the right side)

The surgical procedure was completed with placement of 370-cc bilateral silicone gel implants Figure 3

**Figure 4:**
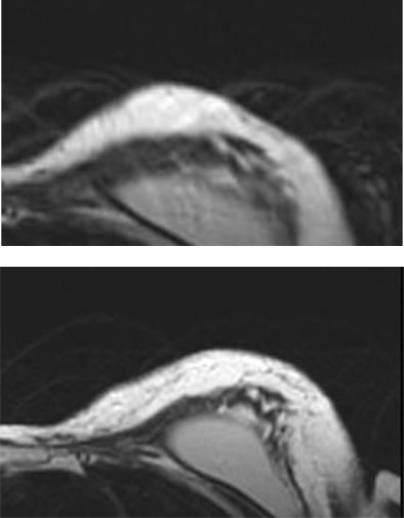
Post-operative evaluation of graft retention by MRI at 3 months

The patient was discharged on post-operative day 1 and had no complications in the post-operative course. The aesthetic result was evaluated during a ten month follow-up after the second procedure. The patient evaluated the result as excellent, the surgeon as good. Graft retention in the mastectomy flap was evaluated with MRI and ultrasound at three months Figure 4.

The oncological follow-up was negative at 17 months for local relapses and systemic metastasis.

## Discussion

Autologous fat grafting has been used to treat soft tissue defects for decades with varying degrees of success. Fat would be an ideal filler given the easy accessibility, lack of immunogenicity and low cost of adipose tissue, but survival of fat grafts has not been predictable [4,5]. Several studies have suggested that the addition of adipose-derived regenerative cells (ADRCs) to fat can lead to increased graft survival [6–9]. The mechanism of this effect is not fully understood but ADRCs are known to differentiate into replacement tissue, express growth factors, which enhance angiogenesis and modulate the inflammatory response favouring healing over scarring [10–13].

In this case report, a new technique is used to enrich a patient's fat with her own ADRCs to create a natural soft tissue filler. Cell-enhanced fat along with a silicone implant is used for breast reconstruction after NASM. The follow-up demonstrates that ADRC-enhanced fat grafting is a feasible option for breast reconstruction, which may lead to a more natural appearance than implant alone.
